# Feeding and growth of the marine heterotrophic nanoflagellates, *Procryptobia sorokini* and *Paraphysomonas imperforata* on a bacterium, *Pseudoalteromonas* sp. with an inducible defence against grazing

**DOI:** 10.1371/journal.pone.0195935

**Published:** 2018-04-13

**Authors:** Jakob Tophøj, Rasmus Dam Wollenberg, Teis Esben Sondergaard, Niels Thomas Eriksen

**Affiliations:** Department of Chemistry and Bioscience, Aalborg University, Aalborg, Denmark; VIT University, INDIA

## Abstract

Heterotrophic marine nanoflagellates are important grazers on bacteria in the water column. Some marine bacteria appear more resistant to grazing than do others. Marine nanoflagellates can be grown in the laboratory in batch cultures fed specific bacterial isolates. In some cultures, the flagellates appear unable to completely deplete the bacterial prey even when the bacterial strain otherwise is an excellent prey. This may indicate that some marine bacteria are able to induce defence mechanisms if they are grazed by nanoflagellates. Four morphologically distinct marine heterotrophic nanoflagellates, of which 3 were still identified as *Procryptobia sorokini* (Kinetoplastea) and one as *Paraphysomonas imperforata* (Chrysophyceae) were isolated from a coastal location along with 3 isolates of the marine bacterium *Pseudoalteromonas* sp. Flagellate growth and grazing on bacterial prey were analysed in batch cultures. *Pseudoalteromonas* was a suitable prey for all 4 flagellate isolates. They grazed and grew on *Pseudoalteromonas* as sole prey with maximal cell-specific growth rates of 0.1–0.25 h^-1^ and gross growth efficiencies of 38–61%. Exposure to dense flagellate cultures or their supernatants did, however, cause a fraction of the *Pseudoalteromonas* cells to aggregate and the bacterium became apparently resistant to grazing. Concentrations of suspended *Pseudoalteromonas* cells were therefore not decreased below 1,700–7,500 cells μL^-1^ by any of the flagellate isolates. These results indicate that *Pseudoalteromonas* sp. can be an excellent prey to marine nanoflagellates but also that is in possession of inducible mechanisms that protect against flagellate grazing.

## Introduction

Heterotrophic nanoflagellates play important roles in marine environments as bacterial grazers. Pelagic nanoflagellates control the concentration of bacteria in the water column [1, 2] and mediate the transfer of bacterial biomass to higher trophic levels of aquatic food webs [3]. Not all bacteria are captured and consumed equally efficient by all flagellates. Most interception feeding nanoflagellates (e.g. kinetoplastids and chrysophytes) seem to prefer larger, non-motile bacterial cells while smaller bacterial cells are preferred by e.g. the filter feeding choanoflagellates [4–6]. Bacteria may use a range of defence mechanisms to protect themselves from flagellate grazing, such as cell surface masking, digestional resistance, toxin production, aggregation, and microcolony formation [7]. Bacteria that are resistant to grazing may gain competitive advantages over other bacteria when grazed by nanoflagellate. In chemostat and mesocosm experiments, grazing resistant bacterial species [8, 9] or grazing resistant phenotypes of otherwise grazing sensitive bacteria [10–12] have dominated bacterial populations when nanoflagellates were also present. Evolutionary adaptations improving grazing resistance of bacterial strains have also been described [13]. It has furthermore been observed that some marine bacteria change physiology in the presence of heterotrophic nanoflagellates, grow in microcolonies, and secrete mucus to protect against grazing [14–16]. Flagellates are recognized by chemosensory mechanisms and the protective responses can be induced by cell free medium or filtrates from flagellate cultures [15, 16]. Grazing resistance may have a metabolic cost and grazing sensitive bacteria tends to become dominating in the absence of flagellate predators [9–11, 13].

Growth and bioenergetics have been investigated in several marine nanoflagellates [17– 29]. Maximal specific growth rates are commonly between 0.1–0.25 h^-1^ at 10–20°C with net growth efficiencies of 15–61% (measured either in units of biomass or cell carbon). The relationship between specific growth rate and concentration of bacterial cells can be described by Monod type saturation kinetics with half saturation constants of 1,300–45,000 bacterial cells per μL [17, 19–21, 23, 29]. These half-saturation constants are higher than the bacterial concentrations (1,000 bacterial cells per μL or less) normally found in marine waters [30] and bacterivorous marine nanoflagellates generally live under food limited conditions. In several studies, however, where flagellates have been grown in batch cultures, have the flagellates stopped taking up bacteria at concentrations of 2–20,000 bacterial cells per μL [19, 23, 28]. We observed the same phenomenon in preliminary batch cultures of marine nanoflagellates fed an isolated marine bacterium, *Pseudoalteromonas* sp. while there seemed not to be a clear relationship between initial and final *Pseudoalteromonas* concentrations. This could suggest that *Pseudoalteromonas* sp. may be able to activate growth independent responses that protect against grazing. Bacteria of the genus *Pseudoalteromonas* are widespread in marine environments [31] and among the bacteria that can readily be isolated from seawater and grown in axenic cultures in the laboratory. These bacteria are believed to be suitable prey for marine nanoflagellates since flagellate grazing apparently maintains the concentration of *Pseudoalteromonas* and other large gram-negative bacteria low in the sea [32]. *Pseudoalteromonas* species are known to secrete anti-bacterial compounds, toxins, extracellular enzymes, and exopolysaccharides, and surface dwelling strains can inhibit growth and settlement of other organisms [31].

If grazing protective mechanisms can be induced also in non-growing marine bacteria, such mechanisms may be found in e.g. *Pseudoalteromonas* species because these bacteria are heavily grazed [32] and in other connections have shown diverse metabolic capabilities [31]. In this paper, we have quantified growth of 4 heterotrophic, interception feeding, marine nanoflagellates feeding on 3 *Pseudoalteromonas* isolates, verified that *Pseudoalteromonas* sp. is excellent prey to these marine flagellates, and observed that *Pseudoalteromonas* indeed appears to be in possession of defence mechanisms that can be induced to protect them from being grazed by the flagellates.

## Materials and methods

### Sampling, isolation, and cultivation of marine nanoflagellates

Seawater (temperature = 8°C, salinity = 22‰) was sampled at a coastal location (public beach area) at Dokkedal, Denmark (56°54'21.2"N 10°16'9.2"E) 5–40 cm below the surface and brought to the laboratory and incubated at 15°C in the dark. Seawater for preparation of growth media was collected at the same location, filtered through 0.22 μm filters and autoclaved after enrichment by 0.01–1 g L^-1^ yeast extract.

Nanoflagellates were propagated by mixing 80 mL of freshly sampled sea water with 20 mL of medium, reaching a final concentration of yeast extract of 0.01 g L^-1^. After 2 weeks of incubation at 15°C in the dark, the cultures were examined for the presence of heterotrophic nanoflagellates under the microscope, diluted in sterile seawater until a flagellate concentration of 1 cell per μL, and 1 μL was transferred into a well in a microtiter plate containing 200 μL of sterile medium containing 0.01 g L^-1^ of yeast extract. This left on average 1 flagellate cell in each well. After 4–5 days if incubation at 15°C, each well was examined for the presence of flagellates under the microscope. Water from wells containing nanoflagellates were again diluted to a flagellate concentration of 1 cell per μL and transferred into a new well repeating the procedure described above. This procedure was repeated at least twice until all flagellates in one well appeared morphologically identical under the microscope (phase contrast, 40x and 60x objectives). Biweekly, the flagellate isolates were transferred into new batch cultures at 15°C in 22‰ autoclaved seawater supplemented by 0.01 g L^-1^ of yeast extract.

### Isolation and cultivation of *Pseudoalteromonas* sp.

Seawater was sampled at Dokkedal, Denmark and diluted in sterile sea water until a bacterial concentration of approximately 10 cells per μL where after 25 μL was spread on petri dishes containing seawater enriched by 1 g L^-1^ of yeast extract and solidified by 10 g L^-1^ agar. The plates were incubated at 15°C for 4–5 days, and individual colonies were transferred to 0.22 μm filtered and autoclaved seawater enriched by 1 g L^-1^ of yeast extract and grown in batch cultures at 15°C. Three of these clonal isolates supported growth of clonal nanoflagellate cultures and were maintained in liquid culture and used as flagellate feed.

### Molecular identification of nanoflagellate and bacterial isolates

Four supposedly clonal nanoflagellate isolates and three clonal bacterial isolates that supported growth of the flagellates were identified by partial 18S rDNA or 16S rDNA sequencing, respectively. Approximately 25 mg of lyophilized biomass was subjected to bead-beating (6000 rpm for 3×5 seconds using 1.4 mm ceramic spheres, 0.1 mm silica spheres, and one 4 mm glass bead) on a Precellys tissue homogenizer (Bertin, France). Samples were cooled on ice before and after the bead beating. Thereafter genomic DNA was extracted by the Qiagen DNeasy Plant Mini Kit (Qiagen, Germany) or the FastDNA^™^ SPIN kit for soil (MP Biomedicals, USA). The DNA regions encoding part of the 18S and 16S rDNA regions were amplified from 25–50 ng genomic DNA template using primer pairs F-566/R-1200, cryso240/ cryso651, and 27F/1492R, respectively (Table B in S1 File). PCR products were generated in 50 μL reaction volumes containing 0,02 U Phusion^™^ High-Fidelity DNA Polymerase (Thermo Scientific, USA), 1× HF Green Buffer, 200 μM dNTP mix, and 0.5 μM forward and 0.5 μM reverse primer. PCR products were subsequently purified with either the QIAquick PCR Purification Kit (Qiagen, Germany) or the QIAquick Gel Extraction Kit (Qiagen, Germany) and outsourced for sequencing at Eurofins Genomics (Eurofins, Germany). A blastN analysis was performed against the non-redundant database at NCBI to identify the relevant taxa [33].

### Grazing experiments

Nanoflagellate isolates were grown in 100 mL batch cultures in conical flasks incubated at 15°C in an orbital shaking incubator operated at 100 RPM. *Pseudoalteromonas* sp. harvested from 1-day old batch cultures that had entered stationary phase served as feed. Initial concentrations of flagellates were 30–70 cells per μL while initial concentrations of *Pseudoalteromonas* sp. were 7,500–30,000 cells per μL. The bacteria were harvested by centrifugation and resuspended in 1 mL 0.22 μm sterile filtered seawater before added to flagellate cultures in order to minimize potential transfer of left-over components from the bacterial growth medium to the flagellate cultures. Flagellate and bacterial cells were simultaneously counted in samples taken from the cultures and used to quantify growth and grazing (see below).

### Determination of cell densities, cell dry weights, yield coefficients, and gross growth efficiencies

Concentrations of nanoflagellates, *c*_*f*_ and bacteria, *c*_*b*_ were determined from microscopically counts in a 0.0025 mm^3^ hemocytometer (Thoma). The concentration of grazing resistant bacterial cells, *c*_*b*,*end*_ was estimated as bacterial concentrations remaining in each culture after flagellate growth has stopped. In bacterial cultures cell concentrations were also followed indirectly from measurements of the optical density at 600 nm, OD_600_ after dilution to OD_600_ values below 0.3.

The concentration of dry biomass of nanoflagellate or bacterial cultures were measured after filtration of culture onto pre-dried and pre-weighed 0.22 μm MF membrane filters (Millipore) and drying at 105°C overnight. Dry weights of individual bacterial cells, *m*_*b*_ were estimated by comparing dry biomass concentrations to bacterial cell concentrations. Dry weights of individual flagellate cells, *m*_*f*_ were estimated the same way, but after subtraction of the dry bacterial biomass concentrations in the cultures.

The yield coefficient, *Y*_*f/b*_ was estimated from slopes of linear regressions of plots of increase of flagellate concentration, *c*_*f*_−*c*_*f*,*0*_. vs. decrease of bacterial cell concentration, *c*_*b*,0_ –*c*_*b*_. The number of bacterial cells needed to produce one nanoflagellate cell, *Y*_*b/f*_ corresponds to *Y*_*f/b*_^*-1*^. Gross growth efficiency, *GGE* was calculated by multiplying *Y*_*f/b*_ values by the ratio between dry flagellate and bacterial cell weights.

### Modelling of cell concentrations and estimation of flagellate performance

Grazing and growth of flagellates were described using the growth model shown in S1 File. The individual equations in the model were discretized and solved numerically using Euler’s solution at time intervals, *Δt* = 0.2 h, starting at *t* = 0, and in the order shown in Table A in S1 File. Maximal clearance rate, *Cl*_*max*_ and ingestion rate, *I*_*max*_ were estimated by fitting Eqs. A-F in S1 File to experimentally determined concentrations of flagellate and bacterial cells in 3 flagellate batch cultures grown on different initial concentrations of non-growing bacterial cells. The numerical differences between measured and calculated cell concentrations were simultaneously minimized in all 3 cultures. The half saturation constant, *k*_*b*_ was found from Eq. H, while the maximal specific growth rate, *μ*_*max*_ of the flagellates was estimated from Eq. E in S1 File setting *I* = *I*_*max*_.

## Results

### Isolation and identification of nanoflagellates and bacterial prey

Four morphologically distinct nanoflagellates (Fig A in S1 File) were isolated and grown in batch cultures. For 3 of the nanoflagellates, blast analyses of their 18S rDNA sequences showed closest sequence similarity (99%) to GenBank ID KF479401.13 identifying them as *Procryptobia sorokini* (Table D in S1 File). These 3 isolates were named *P*. *sorokini* strains G5, B11, and A5, respectively. *P*. *sorokini* strain A5 had a hook shaped flagellum (Fig A in S1 File) and resembled the morphotype *Neobodo* [34]. *Procryptobia sorokini* (syn. *Bodo sorokini*) as well as *Neobodo* sp. belong to the Kinetoplastea, order Bodonida [35], which is a group of motile, interception feeding nanoflagellates that are common members of the heterotrophic picoplankton. The last of the isolates was identified as *Paraphysomonas imperforata* and named strain A2 (99% sequence similarity to GenBank ID KX431470.1). *P*. *imperforata* belongs to the Chrysophyceae [35] and is also a motile bacterivorous predator. The nanoflagellates had dried cell masses between 96 and 219 pg cell^-1^ during growth phases (Table 1).

**Table 1:** Characteristic values of batch cultures of *Procryptobia sorokini* G5, B11, A5 and *Paraphysomonas imperforata* A2, feeding on *Pseudoalteromonas* sp. B2, B3 or B4. Cell dry weights of flagellates, *m*_*f*_, and bacteria, *m*_*b*_, measured during growth and stationary phase, respectively. Yield of flagellates per bacterium taken up, *Y*_*f/b*_, number of bacterial cells needed to produce one flagellate cell *Y*_*b/f*_, and gross growth efficiency, *GGE* were evaluated from Figs 1C, 1D, 2C and 2D, respectively (data from batch cultures of *P*. *imperforata* A2 feeding on *Pseudoalteromonas* sp. B2 in Fig B in S1 File. Maximal clearance, *Cl*_*max*_, and ingestion rates, *I*_*max*_, are estimated by fitting numerical solutions to Eqs. A and D in S1 File to experimentally determined concentrations of flagellate and bacterial cells (Figs 1A, 1b, 2A and 2B). Maximal specific growth rate, *μ*_*max*_, and half saturation constant, *K*_*b*_, are calculated from Eqs. E and H, respectively. Final bacterial concentration, *C*_*b*.*end*_. Three batch cultures were carried out for each combination of flagellate and bacterial isolate.

Flagellate isolate	Bacterial isolate	*m*_*f*_	*m*_*b*_	*Y*_*f/b*_	*Y*_*b/f*_	*GGE*	*Cl*_*max*_	*I*_*max*_	*μ*_*max*_	*K*_*b*_	*C*_*b*.*end*_
		pg per cell	pg per cell				μL cell^-1^ h^-1^	h^-1^	h^-1^	cells per μL	cells per μL
G5	B2	116	4.0	0.021	48	0.61	0.0041	11.7	0.25	2,854	1,700
B11	B4	124	3.6	0.016	61	0.57	0.0064	6.7	0.11	1,127	4,800
A5	B4	219	3.6	0.010	102	0.60	0.0025	20.7	0.20	8,350	4,600
A2	B3	96	3.1	0.015	67	0.46	0.0013	12.6	0.19	9,705	3,400
A2	B2	96	4.0	0.016	64	0.38	0.0017	6.3	0.10	3,615	7,500

At the location at Dokkedal from where the nanoflagellates were isolated, bacterial numbers in the water varied between 200 and 900 cells μL^-1^ between October 2015 and April 2016. Three bacterial isolates, isolated from the same water samples as the nanoflagellates, were identified through partial 16S rDNA sequencing as *Pseudoalteromonas* sp. and named strain B2, B3, and B4, respectively (showing 99% sequence identity to GenBank ID MF061255.1, Table D in S1 File). *Pseudoalteromonas* sp. grew as solitary rod shaped cells, approximately 2 μm in length and with a diameter of 1 μm. Few aggregates of 10 or more cells were, however, also present in growing cultures. Cells harvested from stationary phase of batch cultures grown in 22‰ seawater supplemented with yeast extract had cell masses of 3–4 pg cell^-1^ (Table 1). All 3 *Pseudoalteromonas* sp. isolates were grazed by the 4 nanoflagellates and supported their growth when used as the only feed.

### Growth and grazing

**Fig 1:**
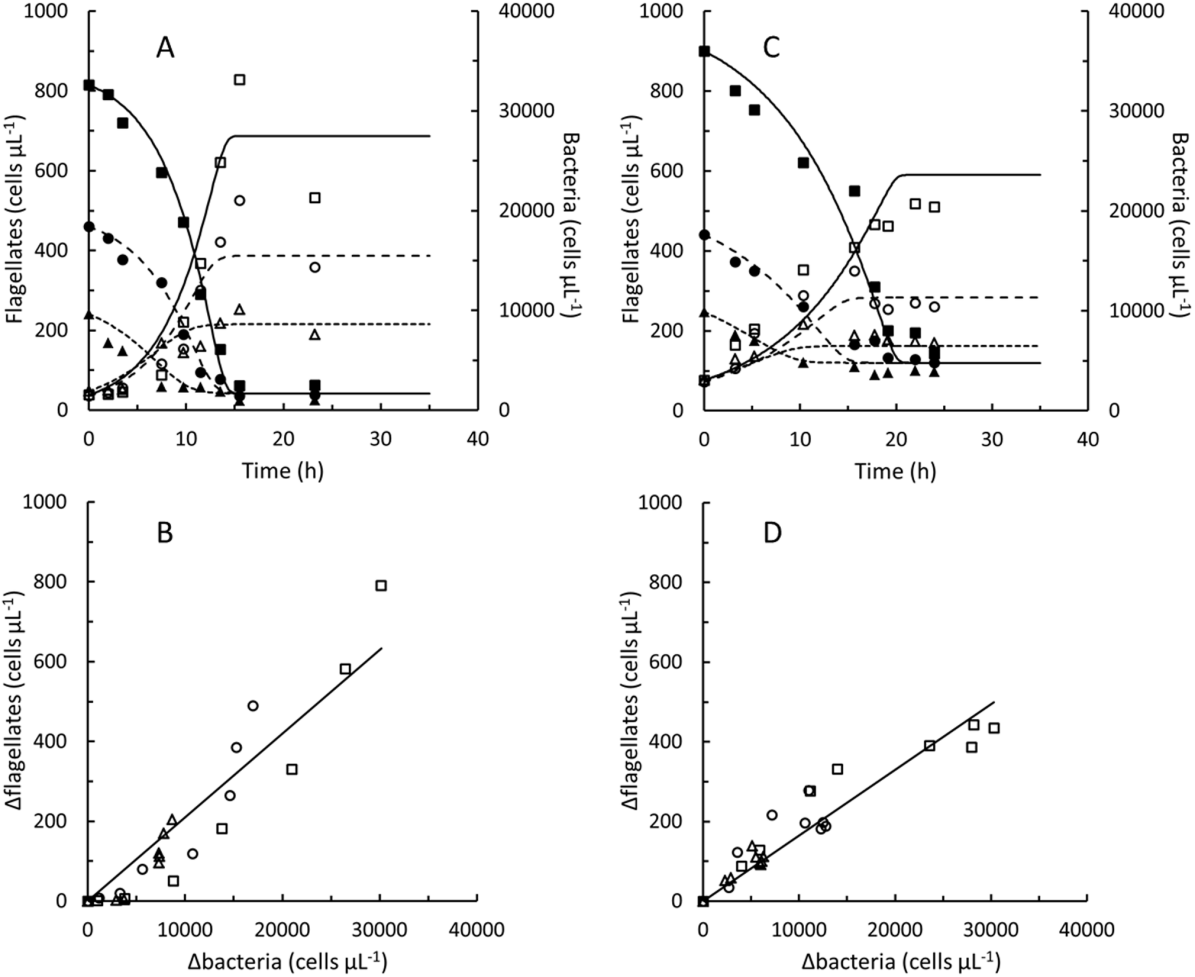
*Procryptobia sorokini*. Batch cultures of *Procryptobia sorokini* G5 feeding on *Pseudoalteromonas* sp. B2 (A and B) and *P*. *sorokini* B11 feeding on *Pseudoalteromonas* sp. B4 (C and D). Concentrations of flagellate cells (open symbols) and bacterial cells (solid symbols) in cultures inoculated at approximately 7,500 (△, ▲), 15,000 (○, ●), and 30,000 (□, ■) *Pseudoalteromonas* sp. μL^-1^, respectively. Curves (A and C) drawn by fitting Eqs. A-F in S1 File to measured concentrations of *Procryptobia sorokini* and *Pseudoalteromonas* sp. Data in S1 Dataset.

**Fig 2:**
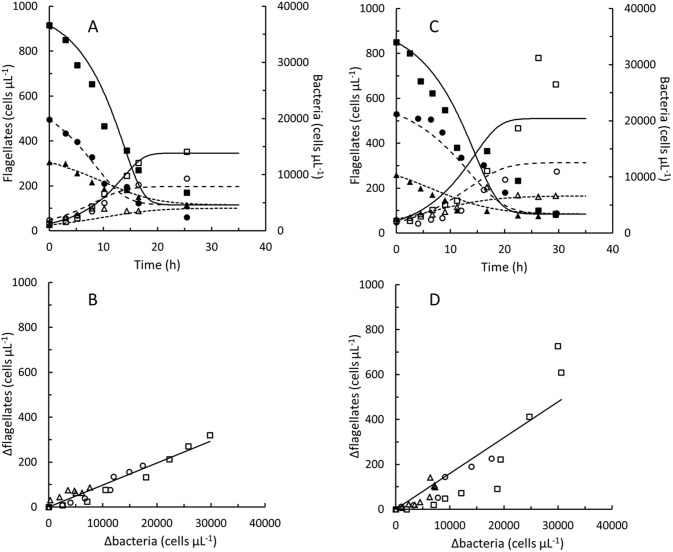
*Procryptobia sorokini* and *Paraphysomonas imperforata*. Batch cultures of *Procryptobia sorokini* A5 feeding on *Pseudoalteromonas* sp. B4 (A and B) and *Paraphysomonas imperforata* A2 feeding on *Pseudoalteromonas* sp. B3 (C and D). Concentrations of flagellate cells (open symbols) and bacterial cells (solid symbols) in cultures inoculated at approximately 7,500 (△, ▲), 15,000 (○, ●), and 30,000 (□, ■) *Pseudoalteromonas* sp. μL^-1^, respectively. Curves (A and C) drawn by fitting Eqs. A-F in S1 File to measured concentrations of flagellates and *Pseudoalteromonas* sp. Data in S1 Dataset.

The 4 nanoflagellates, *P*. *sorokini* G5, B11, A5 and *P*. *imperforata* A2 were grown in batch cultures on different concentrations of *Pseudoalteromonas* sp. (Figs 1 and 2). In control experiments without flagellates, bacterial concentrations decreased by 0–10% during 14 h periods (corresponding to the growth phase of flagellate cultures). The seawater used was therefore too low in nutritional compounds to supported additional cell divisions in *Pseudoalteromonas* sp. (under nutrient sufficient conditions the doubling time of *Pseudoalteromonas* sp. was approximately 1.5 h). In control experiments without addition of *Pseudoalteromonas* sp., flagellate concentrations stayed constant or decreased by up to 30% during 24 h periods. Flagellate growth and decreasing bacterial concentrations were therefore linked to grazing in these experiments. Flagellate concentrations increased in proportion to the decrease in bacterial concentrations (Figs 1 and 2). The yield, *Y*_*f/b*_ varied between 0.010 and 0.021 flagellate cell per ingested *Pseudoalteromonas* cell (Table 1), meaning that between 48 and 102 bacterial cells were ingested to produce one flagellate cell. The estimated gross growth efficiency, *GGE* taken into account the masses of the flagellate as well as the *Pseudoalteromonas* cells were between 38 and 61%. Fairly similar *GGE*’s (24–50%) were found when cell masses were predicted from rough estimates of cell volumes (Table C in S1 File), although cell masses estimated from the cell volumes were lower than the measured ones.

None of the nanoflagellate cultures were able to decrease the concentration of bacterial cells to zero. Irrespectively of the initial bacterial density, 2–5,000 bacteria per μL remained in the cultures when the flagellates entered stationary phase (Figs 1 and 2). Flagellate and bacterial concentrations were modelled by Eqs. A-H in S1 File and maximal clearance rates, *Cl*_*max*_ and ingestion rates, *I*_*max*_ are listed in Table 1, along with maximal specific growth rates, *μ*_*max*_ and half saturation constants, *k*_*b*_. Clearance and ingestion rates predicted from Eqs. B and C in S1 File as function of bacterial concentration are shown in Fig C in S1 File.

### Responses in *Pseudoalteromonas* sp.

**Fig 3:**
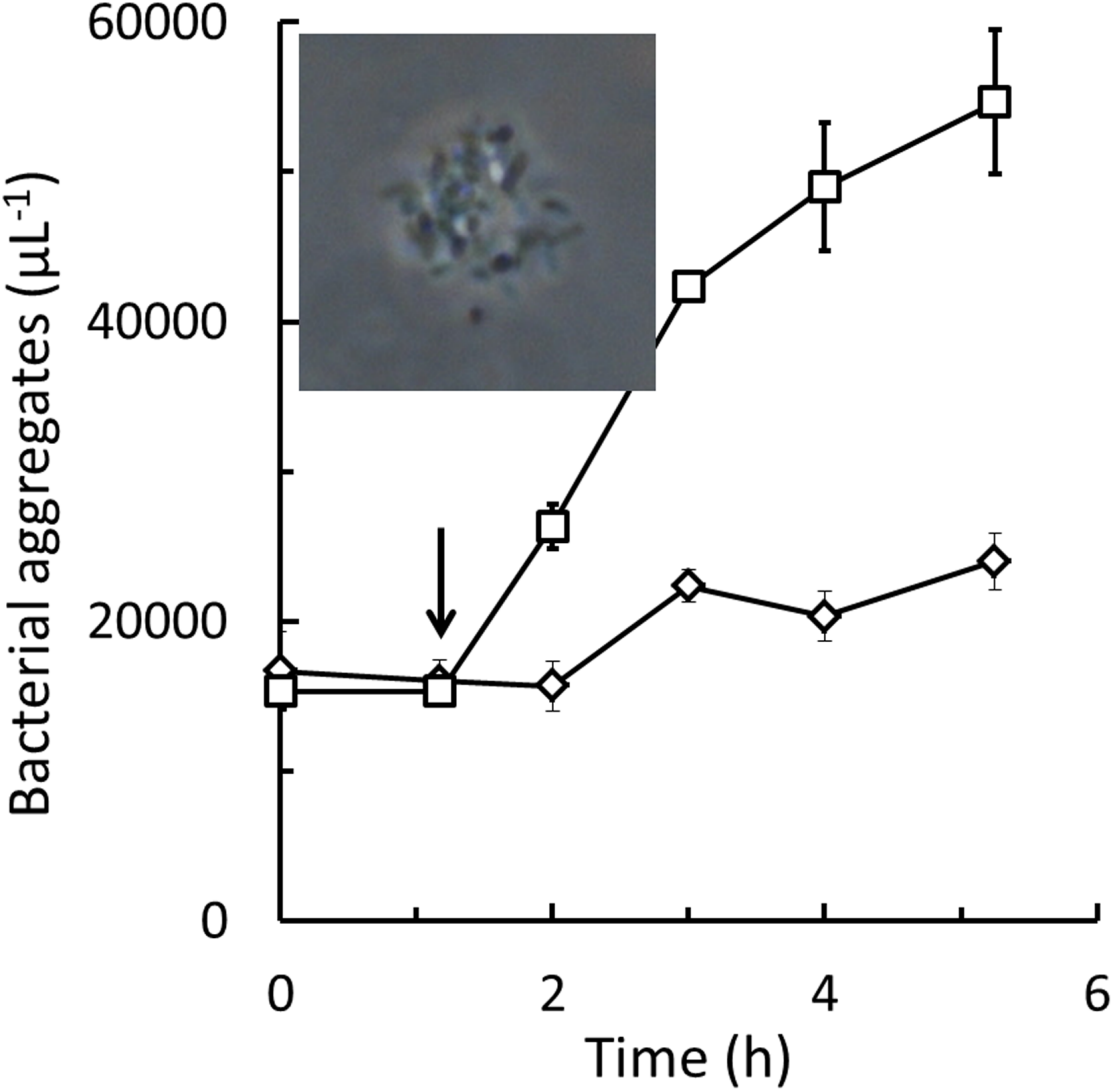
*Pseudoalteromonas*. Concentration of aggregates >10 cells of *Pseudoalteromonas* sp. B2. Arrow marks addition of 10% 0.22 μm sterile filtered culture supernatant from a stationary phase culture of *Procryptobia sorokini* G5 (□) or 10% 0.22 μm sterile filtered seawater (◊). Initial concentrations were 1,000,000 solitary *Pseudoalteromonas* sp. B2 μL^-1^. Inset is micrograph showing aggregated *Pseudoalteromonas* sp. B2 cells viewed under phase contrast.

**Fig 4:**
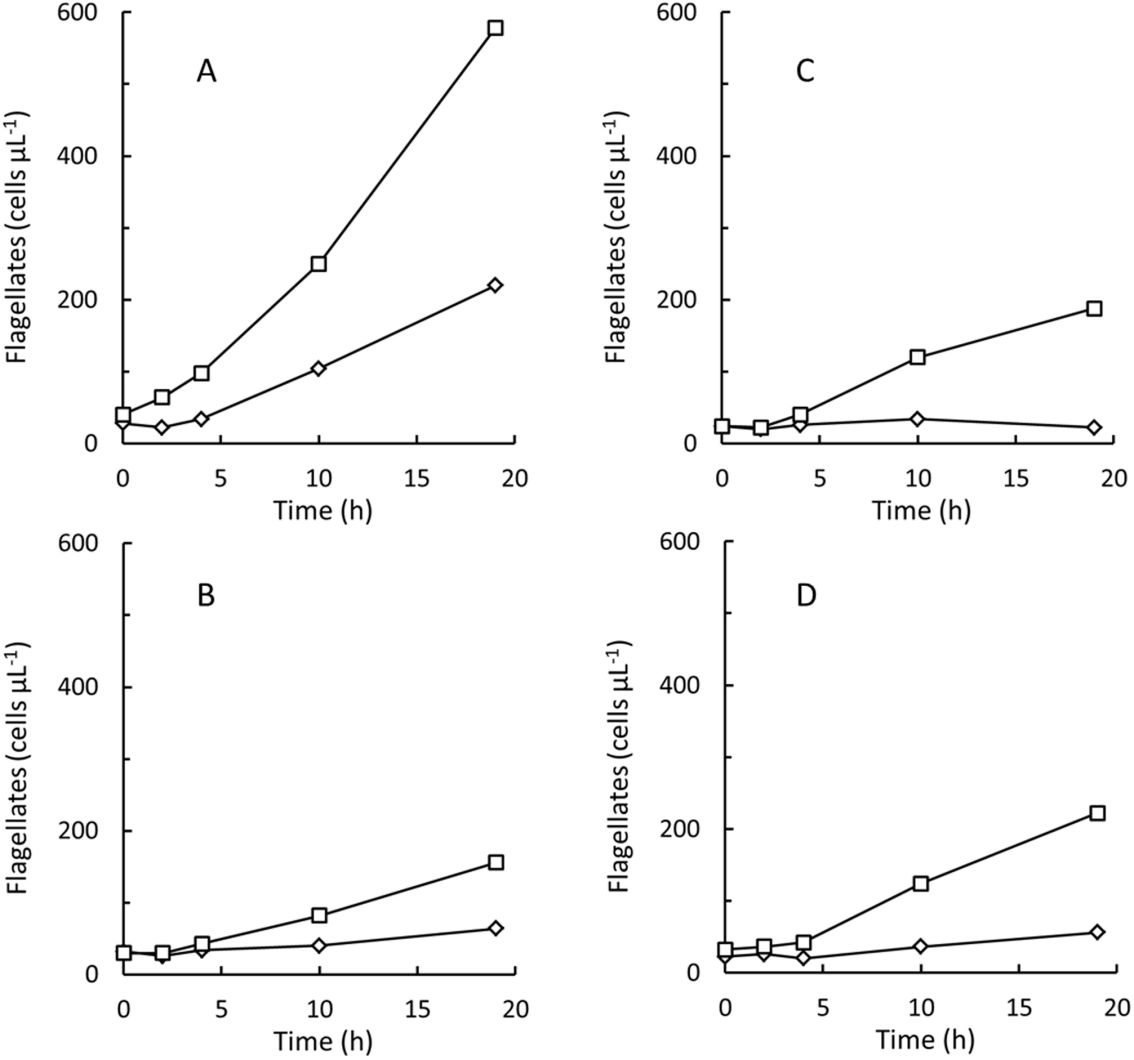
*Procryptobia sorokini* and *Paraphysomonas imperforata*. Batch cultures of *Procryptobia sorokini* G5 fed *Pseudoalteromonas* sp. B2 (A), *P*. *sorokini* B11 fed *Pseudoalteromonas* sp. B4 (B), *P*. *sorokini* sp. A5 fed *Pseudoalteromonas* sp. B4 (C), and *Paraphysomonas imperforata* A2 fed *Pseudoalteromonas* sp. B3 (D). Concentrations of flagellates in cultures added 5% 0.22 μm sterile filtered culture supernatant from a stationary phase culture of a different flagellate isolate (◊) or 5% 0.22 μm sterile filtered seawater (□).

The major fraction of the *Pseudoalteromonas* sp. cells in flagellate cultures remained solitary until the cultures were terminated (bacterial concentrations shown in Figs 1 and 2 represent solitary cells) but at the end of the experiments, a fraction also formed aggregates of 10 or more bacterial cells. Increased aggregation of *Pseudoalteromonas* sp. could be induced also by cell free culture supernatant from the nanoflagellate cultures. Fig 3 shows numbers of bacterial aggregates in *Pseudoalteromonas* sp. B2 diluted in seawater with and without addition of 10% spent supernatant from a stationary phase *P*. *sorokini* G5 culture, sterile filtered through a 0.22 μm syringe filter. After 1 h of incubation, higher numbers of bacterial aggregates were observed after the spent culture supernatant had been added. Increased cell aggregation was likewise observed in *Pseudoalteromonas* sp. B3 and B4 upon addition of 0.22 μm sterile filtered supernatant from cultures of all 4 nanoflagellates. When cell free culture supernatant taken from stationary phase of flagellate batch cultures was added to newly inoculated flagellate cultures, also flagellate growth was inhibited. Fig 4 compares growth of the 4 nanoflagellates in batch cultures with and without addition of 5% 0.22 μm sterile filtered supernatant taken from stationary phase cultures of one of the other flagellate isolates. Bacterial aggregates appeared in all the flagellate cultures to which spent culture supernatant had been added. Finally was it observed, that 0.22 μm sterile filtered supernatant taken from flagellate batch cultures also effected growth of freshly inoculated batch cultures of *Pseudoalteromonas* sp. (Fig D in S1 File).

## Discussion

The 4 nanoflagellates isolated in this study, *Procryptobia sorokini* G5, B11, A5 and *Paraphysomonas imperforata* A2 were all able to graze and grow on *Pseudoalteromonas* sp. as their sole prey. This bacterium seems, however, also able to become resistant to flagellate grazing and was never completely depleted despite the high concentrations of flagellates. Cell aggregation may be part of the mechanism that protects *Pseudoalteromonas* sp. against flagellate grazing but since most of the *Pseudoalteromonas* cells remained solitary, cell aggregation may not be the only protective mechanism in their possession. Since addition of cell free culture supernatant from flagellate cultures was sufficient to induce aggregation of *Pseudoalteromonas* cells, this bacterium must be able to detect flagellates by a chemosensory mechanism. Our data suggest that *Pseudoalteromonas* can be excellent prey for flagellates but also that the presence of flagellates rapidly can induce growth independently defence mechanisms against grazing in *Pseudoalteromonas* sp.

*Pseudoalteromonas* are gram-negative, rod shaped bacteria. They are large bacterial cells [32] with dry weights of 3–4 pg cell^-1^ (Table 1). They therefore seem to fulfil the several criteria for being suitable prey to interception feeding nanoflagellates [4–6, 17, 18, 22, 27]. Specific growth rates ranged from 0.1–0.25 h^-1^ (Table 1), which is within the expected range for heterotrophic nanoflagellates [17, 19, 22, 24, 29]. In *P*. *imperforata* A2, the maximal clearance rates of 1.3 and 1.7 nL per cell per h agreed excellently with earlier measurement of 1.55 nL per cell per h [36] while higher *Cl*_*max*_ values have been found in other heterotrophic nanoflagellates [17]. Maximal ingestion rates ranged from 6 to 21 bacterial cells per cell per h. These values are low compared to what have been found in earlier studies. E.g. has *I*_*max*_ previously been estimated to 62–103 cells per cell per h in *P*. *imperforata* [21, 24] while 6 different nanoflagellates had *I*_*max*_ values of 27–254 cells per cell per h [17]. Clearance and ingestion rates show, however, considerable variations among nanoflagellates [22] and the low ingestion rates were probably related to the large cell size of *Pseudoalteromonas* sp. Gross growth efficiencies (*GGE*’s) may therefore represent at better foundation for comparing the performances of different flagellates feeding on different prey. The *GGE*’s of the 4 nanoflagellates feeding on *Pseudoalteromonas* sp. were between 38 and 61% (Table 1). These *GGE* estimates are high but within the range observed also for other heterotrophic nanoflagellates [17–20, 22, 23, 25–27]. The *GGE*’s of *P*. *imperforata* A2 of 36–46% (Table 1) were also within the range of 15–54% (based on either mass or bio volume) previously reported for this species [21, 24, 29]. Changes in flagellate cell size during the final cell generation [17, 20] or cryptic bacterial growth stimulated by re-mineralising of nutrients during the experiments [23] may, however, potentially have resulted in too high *GGE* estimates. Still, the high *GGE*’s in combination with the high *μ*_*max*_ indicate that *Pseudoalteromonas* can be an excellent prey to all 4 nanoflagellate isolates. The 4 nanoflagellates and the 3 *Pseudoalteromonas* strains were isolated from the same waters. Potentially, it may therefore be advantageous for *Pseudoalteromonas* sp. to be in possession of inducible defensive mechanisms against flagellates, although the defensive reactions observed in this study appeared at artificially high concentrations of flagellate and bacterial cells.

When flagellate grazing had decreased the concentration of *Pseudoalteromonas* sp. to 2–5,000 cells per μL grazing stopped and bacterial and flagellate concentrations became stable (Figs 1 and 2). The decrease in concentrations of *P*. *sorokini* G5 after 15 h (Fig 1A) is maybe a result of cannibalism [37]. Grazing and growth did not resume if additional *Pseudoalteromonas* sp. was added (data not shown). Inhibition of newly inoculated flagellate cultures by spent culture supernatant (Fig 4) demonstrates that it was not the high flagellate concentrations that were growth-inhibiting. Because grazing stopped at fairly high bacterial concentrations, *K*_*b*_ values could only be estimated indirectly from Eq. H. The accuracy of these estimates may be hampered by the fact that the grazing-resistant bacterial sub-population, *c*_*b*,*end*_ was modelled as being constant (Eq. F in S1 File) despite the protective mechanisms in *Pseudoalteromonas* sp. rather appeared to be inducible (Figs 3 and 4). Still, the estimated *K*_*b*_ values of 1,100–9,700 bacterial cells per μL (Table 1) are within the range of *K*_*b*_ values found also in other marine nanoflagellates [17, 19, 20, 22, 24, 29].

Bacterial concentrations of 2–5,000 cells per μL at which flagellate growth stopped cannot be expected to represent lower limits for nanoflagellate growth and grazing, also the same has been observed also in cultures of other heterotrophic marine nanoflagellates [19, 23, 28], but may rather be a result of the induction of protective mechanisms in the bacterial prey. The *K*_*b*_ values (Table 1) were of the same order of magnitude as the lowest bacterial cell concentrations in the cultures (Figs 1 and 2). If no protective mechanisms had been induced in *Pseudoalteromonas* sp., ingestion and specific growth rates of the flagellates should expectedly have proceeded at half their maximal values at the bacterial concentrations at which grazing and growth actually stopped. In comparison to bacterial concentrations in seawater [30], 2–5,000 bacteria per μL are a high. We found e.g. only 200–900 bacterial cells per μL at the location where the nanoflagellates were isolated during the winter period from October to March while a second study counted 2–4,000 bacterial cells per μL at a nearby location during August and September [1]. Data from earlier studies do also show that nanoflagellate batch cultures in some cases have been able to decrease bacterial numbers to levels at or below 1,000 cells per μL [17, 29, 38].

Aggregation could be one mechanism used by *Pseudoalteromonas* sp. to protect against flagellate grazing [7] as partial aggregation of non-growing cells could be induced in all 3 *Pseudoalteromonas* by supernatant from all the isolated nanoflagellates, despite *P*. *imperforata* taxonomically is unrelated to *P*. *sorokini*. Aggregation is probably not the only protective mechanism in *Pseudoalteromonas* sp. since the concentration of solitary bacterial cells remained above the *K*_*b*_ values after flagellates entered stationary phase (Figs 1 and 2). Aggregation may also not be particularly efficient in natural waters if cell concentrations are low. Although bacterial aggregates are avoided by some nanoflagellates they may actually be preferred by others [39]. This could be one reason for at bacterium to use more than one protective mechanism. Protective mechanisms impose a metabolic load [9–11, 13]. Inducible grazing mechanisms may therefore be way to preserve the ability of *Pseudoalteromonas* sp. to compete with other bacteria when grazing pressures are low. The results of this study therefore suggest that *Pseudoalteromonas* sp. may indeed be an excellent prey to marine nanoflagellates but also that inducible defence mechanisms can protect it against flagellate grazing.

## Supporting information

S1 Dataset Contains data (i.e. measured and modelled concentrations of nanoflagellates and *Pseudoalteromonas* sp.) from flagellate batch cultures presented in Figs 1, 2 and Fig B in S1 File. (XLSX)

S1 File Describes the growth model used to evaluate parameter describing clearance, ingestion and growth in the heterotrophic marine nanoflagellates, as well as 2 tables and 4 figures. Table A The order in which Eqs. A-E in the growth model are evaluated to model the concentrations of flagellates and bacterial prey cells. Table B DNA primers used in this study of the identification of the nanoflagellates and the bacterium *Pseudoalteromonas* sp. Table C Gross growth efficiencies estimated from cell dimensions. Table D Partial 18S rDNA or 16S rDNA sequences used to identify flagellates and *Pseudoalteromonas* sp. Fig A Micrographs of nanoflagellate isolates fixed in Lugol’s solution. Fig B Batch cultures of *Paraphysomonas imperforata* A2 feeding on *Pseudoalteromonas* sp. B2. Fig C Predicted clearance and ingestion rates as function of bacterial prey concentration. Fig D Batch cultures of *Pseudoalteromonas* sp. grown with and without addition of sterile supernatant from a *Procryptobia sorokini* culture.
(PDF)
