# Supplementary material for: Feeding and growth of the marine heterotrophic nanoflagellates, Procryptobia sorokini and Paraphysomonas imperforata on a bacterium, Pseudoalteromonas sp. with an inducible defence against grazing
Source: PLoS One. 2018 Apr 13;13(4):e0195935. doi: 10.1371/journal.pone.0195935 (PMC5898755; doi:10.1371/journal.pone.0195935)
Supplement: S1 File — Table A The order in which Eqs. A-E in the growth model are evaluated to model the concentrations of flagellates and bacterial prey cells. Table B DNA primers used in this study of the identification of the nanoflagellates and the bacterium Pseudoalteromonas sp. Table C Gross growth efficiencies estimated from cell dimensions. Table D Partial 18S rDNA or 16S rDNA sequences used to identify flagellates and Pseudoalteromonas sp. Fig A Micrographs of nanoflagellate isolates fixed in Lugol’s solution. Fig B Batch cultures of Paraphysomonas imperforata A2 feeding on Pseudoalteromonas sp. B2. Fig C Predicted clearance and ingestion rates as function of bacterial prey concentration. Fig D Batch cultures of Pseudoalteromonas sp. grown with and without addition of sterile supernatant from a Procryptobia sorokini culture. (PDF) [file pone.0195935.s002.pdf]

Supporting Information

Feeding and growth of the marine heterotrophic nanoflagellates, *Procryptobia sorokini* and *Paraphysomonas imperforata* on a bacterium, *Pseudoalteromonas* sp. with an inducible defence against grazing

Jakob Tophøj, Rasmus Dam Wollenberg, Teis Esben Sondergaard, Niels Thomas Eriksen
Department of Chemistry and Bioscience, Aalborg University,
Fredrik Bajers Vej 7H, DK-9220 Aalborg, Denmark

Growth model

In batch cultures of interception feeding nanoflagellates, grown on non-growing bacterial cells, the concentration of bacterial prey cells, c_b decreases by a rate which depends on the concentration of flagellate cells, c_f and the rate by which each flagellate ingests bacteria, I

$$-\frac{dc_b}{dt} = I \cdot c_f \tag{A}$$

The ingestion rate depends on the rate by which each flagellate is able to clear the water for bacterial cells, known as the clearance rate, Cl

$$I = Cl \cdot c_b \tag{B}$$

Since bacterial cells are ingested one by one by interception feeding nanoflagellates, and ingestion of each bacterial cell is associated with a certain processing time [1], a maximal ingestion rate, I_{max} must exist. The clearance rate will therefore decrease when the ingestion rate is high and hyperbolic type relationships between Cl and c_b have been observed before in different nanoflagellates [2, 3]. Such hyperbolic relationships can be described as

$$Cl = Cl_{max} \cdot \frac{I_{max}}{I_{max} + (Cl_{max} \cdot c_b)} \tag{C}$$

where Cl_{max} and I_{max} describe maximal clearance and ingestion rates, respectively.

The concentration of flagellate cells, c_f will increase at a rate that depends on their concentration and their specific growth rate, μ

$$\frac{dc_f}{dt} = \mu \cdot c_f \tag{D}$$

If growth is balanced, the specific growth rate is determined by the yield by which bacterial cells are converted into new flagellate cells, $Y_{f/b}$ multiplied by the ingestion rate

$$\mu = Y_{f/b} \cdot I \tag{E}$$

In our batch experiments, the bacterial cells were never depleted by flagellate grazing since exposure to flagellates seemed to induce a defence response in the bacteria. Since the term c_b represents only the concentration of bacterial cells that are available for flagellate grazing, c_b was estimated as

$$c_b = c_{b,total} - c_{b,end} \tag{F}$$

where $c_{b,total}$ and $c_{b,end}$ are the total concentration of bacterial cells at a given time and at the end of the flagellate growth and feeding phase, respectively.

Monod type saturation kinetics has previously been used to describe ingestion of bacteria by marine nanoflagellates and their growth [4-6]. If Eq. C is inserted into Eq. B and the right hand expression is rearranged

$$I = Cl_{max} \cdot \frac{I_{max} \cdot c_b}{I_{max} + (Cl_{max} \cdot c_b)} = \frac{I_{max} \cdot c_b}{\frac{I_{max}}{Cl_{max}} + c_b} = \frac{I_{max} \cdot c_b}{K_b + c_b} \tag{G}$$

it is seen that Eq. C is in compliance with Monod type saturation kinetics.

The half-saturation constant, K_b cannot be directly observed if part of the bacterial population becomes resistant to grazing, but can be evaluated as [4]

$$K_b = \frac{I_{max}}{Cl_{max}} \tag{H}$$

and represents the concentration of non-grazing resistant bacteria at which the flagellates attain half their maximal ingestion rate. Eq. C predicts that the clearance rate will be maximal at bacterial prey densities close to zero while Eq. G predicts maximal ingestion rate at high, saturating bacterial prey concentrations.

Table A. The order in which Equations A-E were repeatedly evaluated to model the concentrations of flagellates, c_f and bacterial prey cells, c_b in batch cultures are shown in the table below. Subscripts, *t* and *t+Δt* indicate if values refer to present time, *t* or a time-step Δt = 0.1 h ahead in time, respectively. Symbols otherwise as described in text.

Step	Variable	Equation	Calculation
1	Clearance rate	(C)	$Cl_t = Cl_{max} \cdot \frac{I_{max}}{I_{max} + (Cl_{max} \cdot c_{b,t})}$
2	Ingestion rate	(B)	$I_t = Cl_t \cdot c_{b_t}$
3	Flagellate Specific growth rate	(E)	$\mu_t = Y_{f/b} \cdot I_t$
4	Bacterial prey concentration	(A)	$c_{b,t+\Delta t} = c_{b_t} - I_t \cdot c_{f,t} \cdot \Delta t$
5	Flagellate concentration	(D)	$c_{f,t+\Delta t} = c_{f,t} + \mu_t \cdot c_{f,t} \cdot \Delta t$

Table B. DNA primers used in this study of the identification of the nanoflagellates *Procryptobia sorokini* G5, B11, and A5 (Bodonida) and *Paraphysomonas imperforata* A2 (Chrysophyceae) [7], and the bacterium *Pseudoalteromonas* sp. B2, B3, B4 [8].

Primer ID	Primer sequence 5´→3´	Isolate
F-566	CAGCAGCCGCGGTAATTCC	*P. sorokini* G5, B11, A5<br>*P. imperforata* A2
R-1200	CCCGTGTTGAGTCAAATTAAGC	
cryso240	GGAAACCAATGCGGGGCAAC	*P. imperforata* A2
cryso651	CTATTTTGCTCACAGTAAATGACGAG	
27F	AGAGTTTGATCMTGGCTCAG	*Pseudoalteromonas* sp. B2, B3, B4
1492R	GGYTACCTTGTTACGACTT	

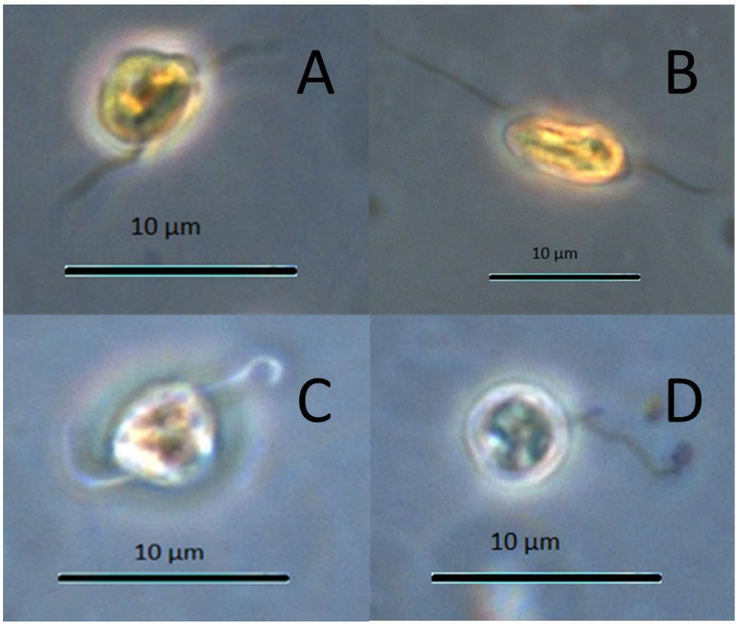

Fig A. *Procryptobia sorokini* and *Paraphysomonas imperforata*. Micrographs of nanoflagellate isolates fixed in Lugol's solution and viewed under phase contrast. A. *Procryptobia sorokini* G5. B. *P. sorokini* B11. C. *P. sorokini* A5. D. *Paraphysomonas imperforata* A2.

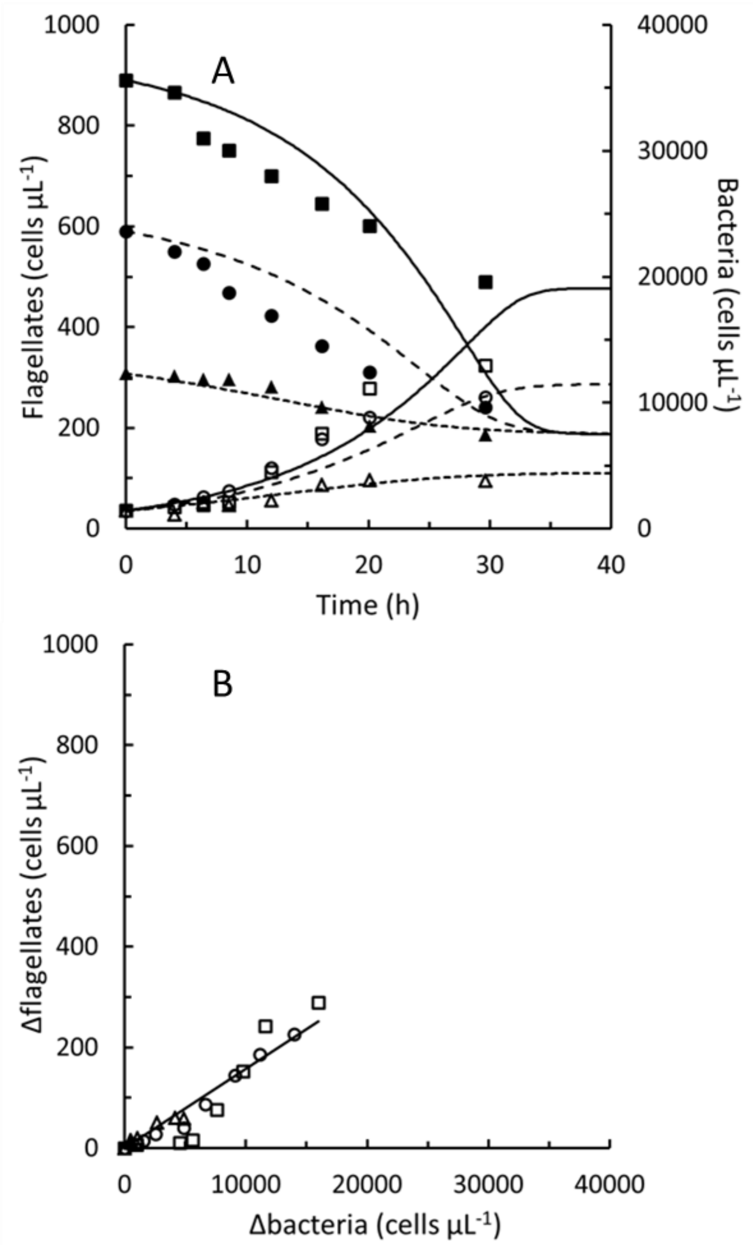

Fig B. *Paraphysomonas imperforata*. Batch cultures of *Paraphysomonas imperforata* A2 feeding on *Pseudoalteromonas* sp. B2. Concentrations of flagellate cells (open symbols) and bacterial cells (solid symbols) in cultures inoculated at approximately 7,500 (△, ▲), 15,000 (○, ●), and 30,000 (□, ■) *Pseudoalteromonas* sp. µL^{-1}, respectively. Curves (A) drawn by fitting Eqs. A-F to measured concentrations of *P. imperforata* and *Pseudoalteromonas* sp. Data in S1 Dataset.

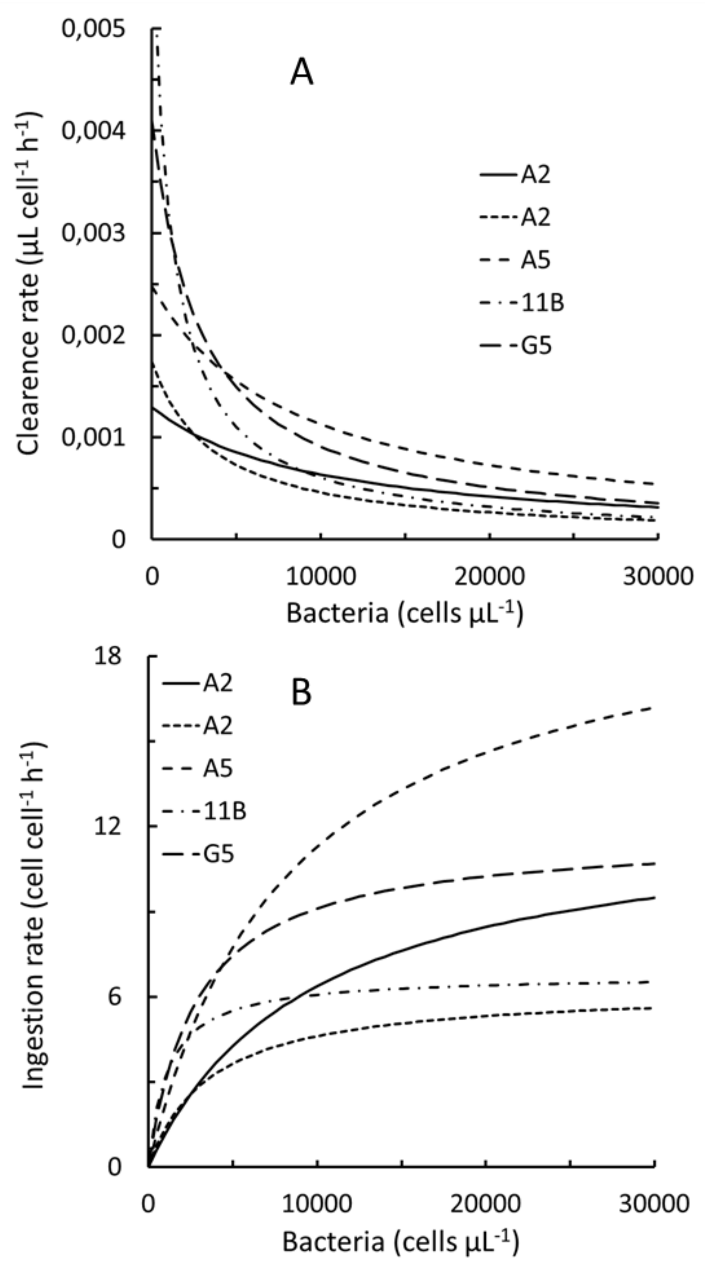

Fig C. *Procryptobia sorokini* and *Paraphysomonas imperforata*. Predicted clearance (A) and ingestion rates (B) as function of bacterial prey concentration in *Paraphysomonas imperforata* A2 feeding on *Pseudoalteromonas* sp. B2 and B3, *Procryptobia sorokini* B11 and A5 feeding on *Pseudoalteromonas* sp. B4, and *P. sorokini* G5 feeding on *Pseudoalteromonas* sp. B2 modelled by Eqs. B and C using the parameters Cl_{max} and I_{max} from Table 1.

Table C. Gross growth efficiencies, *GGE* calculated from rough estimates of cell volumes of flagellates, V_f, and *Pseudoalteromonas* sp., V_b, (flagellate cell volumes calculated as a spherical body, diameter = 6 µm, *Pseudoalteromonas* sp. cell volume calculated as a cylinder, diameter = 1 µm, length = 2 µm). Cell masses of flagellates, m_f, and bacteria, m_b estimated using a density of 1.1 g cm^{-3} and 30% dry weight [9]. Yields of flagellates per bacterium taken up, $Y_{f/b}$ from Table 1.

Flagellate isolate	Bacterial isolate	V_f	V_b	m_f	m_b	$Y_{f/b}$	*GGE*
		$µm^3$	$µm^3$	pg per cell	pg per cell		
G5	B2	150	6	50	2	0.021	0.50
B11	B4	150	6	50	2	0.016	0.38
A5	B4	150	6	50	2	0.010	0.24
A2	B3	150	6	50	2	0.015	0.36
A2	B2	150	6	50	2	0.016	0.39

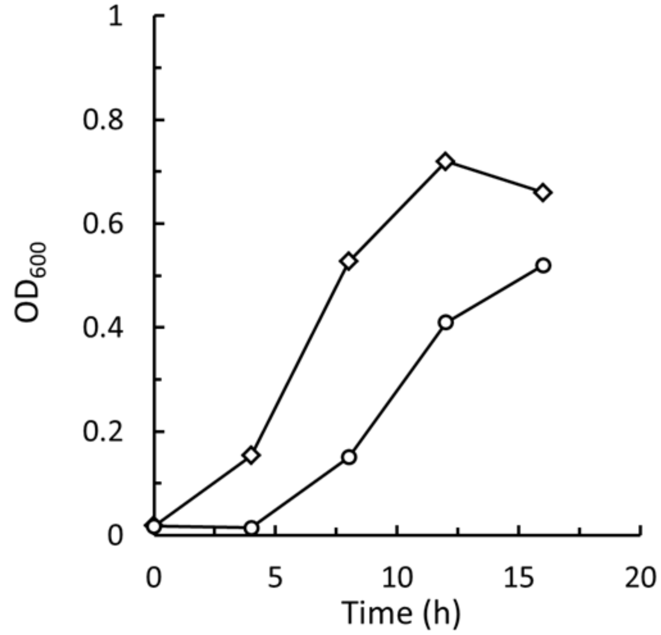

Fig D. ***Pseudoalteromonas.*** Batch cultures of *Pseudoalteromonas* sp. grown in 22‰ seawater enriched with 1 g L^{-1} yeast extract. At t = 1 h one culture was topped up with 5% sterile seawater (◇) and one culture topped up with 5% sterile supernatant taken from a stationary phase culture of a *Procryptobia sorokini* G5 batch culture (○).

Table D. Partial 18S rDNA or 16S rDNA sequences used to identify flagellates and *Pseudoalteromonas* sp.

Strain designation	Clipped Nucleotide Sequence	Best hit	% Identity	E-value
**G5**	TTAAGGGTTCGTAGTTGAATTGAGGGTTGCTCAGATT<br>GGCGGGTTGTCCTCCGGGGCCCCGCCGCTTGCAACTC<br>GCGAACATTCGAAACAAGTAGCACGGGAGCGAATTT<br>CCCTCCGAACACCGCGTGCAACGCACGCAGGAGGGC<br>GCCCGTGATCTTTACTGTGATTAAAAAAGCGTGACCA<br>AAGCAGTCATCCGACATGAATTTCCAAGCATGGGATA<br>ACATCTGTACCTTCGGGACTTCCGTTGGCTTTTGTTGG<br>TTTTAAGGGTCTTAAGGAGATTAAGGCGCCGCGCGA<br>CCCACCAAGGAACGTTAAAACTTCCGAGGTGACCCGG<br>GCAACCGGTTCGCGGCGCTCAAAATGAAGGAGGGTT<br>GTCGGGGACGAACGTACTCGCGCGTGAGAGGTGAAA<br>TTCATAGACCGCGCGAAGACGAACAACAGCGAAGGC<br>ATTCGTCAAGGATACCTTCCTCAATCAAGAACCAAAG<br>TGTGGGGATCGAAGATGATTAGAGACCATTGTAGTC<br>CACGCCACAAACGATGACACCCATGAATTGGGGAAC<br>ATTTGGTTGCGGCCGGCGCGGGCTTGCCCGTTGTCCG<br>GGTTGTGCCATACAACAAATTTACGTGCAGATCCGGG<br>GCCCCCTTCACGGGGGGCTTTAACGTGCATAT	*Procryptobia sorokini*<br><br>GenBank ID KF479401.1	99 %	0.0
**B11**	TTGCTGTTAAGGGTTCGTAGTTGAATTGAGGGTTGCT<br>CAAGATTGGCGGGTTGTCCTCCGGGGCCCCGCCGCTT<br>GCAACTCGCGAACATTCGAAACAAGTAGCACGGGAG<br>CGAATTTCCCTCCGAACACCGCGTGCAACGCACGCAG<br>GAGGGCGCCCGTGATCTTTACTGTGATTAAAAAAGCG<br>TGACCAAAGCAGTCATCCGACATGAATTTCCAAGCAT<br>GGGATAACATCTGTACCTTCGGGACTTCCGTTGGCTT<br>TTGTTGGTTTTAAGGGTCTTAAGGAGATTAAGGCGCC<br>GCGCGACCCACCAAGGAACGTTAAAACTTCCGAGGT<br>GACCCGGGCAACCGGTTCGCGGCGCTCAGAATGAAG<br>GAGGGTTGTCGGGGACGAACGTACTCGCGCGTGAGA<br>GGTGAAATTCATAGACCGCGCGAAGACGAACAACAG<br>CGAAGGCATTCGTCAAGGATACCTTCCTCAATCAAGA<br>ACCAAAGTGTGGGGATCGAAGATGATTAGAGACCAT<br>TGTAGTCCACGCCACAAACGATGACACCCATGAATTG<br>GGGAACATTTGGTTGCGGCCGGCGCGGGCTTGCCCG<br>TTGTCCGGTTGTGCCATACAACAAATTTACGTGCAGA<br>TCCGGGGCCCCCTTCACGGGGGGCTTTAACGTGGATA<br>TCCTCAGCACGTTTTCTTTC	*Procryptobia sorokini*<br><br>GenBank ID KF479401.1	99 %	0.0
**A5**	TAACGCTGTTGCTGTTAAGGGTTCGTAGTTGAATTGA<br>GGGTTGCTCAAGATTGGCGGGTTGTCCTCCGGGGCC<br>CCGCCGCTTGCAACTCGCGAACATTCGAAACAAGTAG<br>CACGGGAGCGAATTTCCCTCCGAACACCGCGTGCAAC<br>GCACGCAGGAGGGCGCCCGTGATCTTTACTGTGATTA<br>AAAAAGCGTGACCAAAGCAGTCATCCGACATGAATTT<br>CCAAGCATGGGATAACATCTGTACCTTCGGGACTTCC<br>GTTGGCTTTTGTTGGTTTTAAGGGTCTTAAGGAGATT<br>AAGGCGCCGCGCGACCCACCAAGGAACGTTAAAACT<br>TCCGAGGTGACCCGGGCAACCGGTTCGCGGCGCTCA<br>GAATGAAGGAGGGTTGTCGGGGACGAACGTACTCGC<br>GCGTGAGAGGTGAAATTCATAGACCGCGCGAAGACG<br>AACAACAGCGAAGGCATTCGTCAAGGATACCTTCCTC<br>AATCAAGAACCAAAGTGTGGGGATCGAAGATGATTA	*Procryptobia sorokini*<br><br>GenBank ID KF479401.1	99 %	0.0

	GAGACCATTGTAGTCCACGCCACAAACGATGACACCC<br>ATGAATTGGGGAACATTTGGTTGCGGCCGGCGCGGG<br>CTTGCCCGTTGTCCGGTTGTGCCATACAACAAATTTAC<br>GTGCAGATCCGGGGCCCCCTTCACGGGGGGGCTTTA<br>ACGTGGGATATCCTCAGCACGTTTTCTTTTCCTTCACG<br>CGAAAGCTTTGAGGTTATAGTCTCAGGGGGGAGTAC<br>GTTCGCAAGAGTGAAACTTAAAGAAATTGACGGAAT<br>GGCACCACAAGACGTGGAGCGTGCGGCTATCTT			
**A2**	CTGATGCCAGACGCGCTCTCCCCGAGGATGGACGCA<br>GAGACCAGGTGCACACCCCGTGAGGGGCGGACCGGT<br>CGCCACGACCAGAAATTCAACTACGAGCTTTTTAACT<br>GCAACAACTTTAGTATACGCTATTGGAGCTGGAATTA<br>CCGCGGCTGCTGGCACCAGACTTGCCCTCCAATTGAT<br>CCTCGATAAGGGATTTAAATTGTTCTCATTCCAATTGC<br>CAGACTTAAAAAAGCCCGGCATTGTTATTTATTGTCAC<br>TACCTCCCTGTGTCAGGATTGGGTAATTTACGCGCCT<br>GCTGCCTTCCTTGGATGTGGTAGCCGTTTCTCAGGCTC<br>CCTCTCCGGAATCGAACCCTAATTCTCCGTTACCCGTT<br>AAAGCCATGGTAGGCCAATACCCTACCATCCAAAGCT<br>GATAGGGCAGAAACTTGAATGATGCATCGATCCGAA<br>GATCGATCCGAAAGTTATTATGAATCACCTGAATCCG<br>GGTTGCCCCGCATTGGTTTCCC	*Paraphysomonas imperforate*<br><br>GenBank ID KX431470.1	99 %	0.0
**B2**	TGGTAACGTCCTCCCGAGGGTTAGACTATCTACTTCT<br>GGAGCAACCCACTCCCATGGTGTGACGGGCGGTGTG<br>TACAAGGCCCGGGAACGTATTCACCGCGTCATTCTGA<br>TACGCGATTACTAGCGATTCCGACTTCATGGAGTCGA<br>GTTGCAGACTCCAATCCGGACTACGACGCACTTTAAG<br>TGATTCGCTTACCTTCGCAGGTTCGCAGCACTCTGTAT<br>GCGCCATTGTAGCACGTGTGTAGCCCTACACGTAAGG<br>GCCATGATGACTTGACGTCGTCCCCACCTTCCTCCGGT<br>TTATCACCGGCAGTCTCCTTAGAGTTCTCAGCATTACC<br>TGCTAGCAACTAAGGATAGGGGTTGCGCTCGTTGCG<br>GGACTTAACCCAACATCTCACAACACGAGCTGACGAC<br>AGCCATGCAGCACCTGTATCAGAGTTCCCGAAGGCAC<br>CAAACCATCTCTGGTAAGTTCTCTGTATGTCAAGTGTA<br>GGTAAGGTTCTTCGCGTTGCATCGAATTAAACCACAT<br>GCTCCACCGCTTGTGCGGGCCCCCGTCAATTCATTTG<br>AGTTTTAACCTTGCGGCCGTACTCCCCAGGCGGTCTA<br>CTTAATGCGTTAGCTTTGAAAAACAGAACCGAGGCTC<br>CGAGCTTCTAGTAGACATCGTTTACGGCGTGGACTAC<br>CGGGGTATCTAATCCCGTTTGCTCCCCACGCTTTCGTA<br>CATGAGCGTCAGTGTTGACCCAGGTGGCTGCCTTCGC<br>CATCGGTATTCCTTCAGATCTCTACGCATTTCACCGCT<br>ACACCTGAAATTCTACCACCCTCTATCACACTCTAGTT<br>TGCCAGTTCGAAATGCAGTTCCCAGGTTGAGCCCGGG<br>GCTTTCACATCTCGCTTAACAAACCGCCTGCGTACGCT<br>TTACGCCCAGTAATTCCGATTAACGCTCGCACCCTCCG<br>TATTACCGCGGCTGCTGGCACGGAGTTAGCCGGTGCT<br>TCTTCTGTCAGTAACGTCACAGCTAGCAGGATTAACT<br>ACTAACCTTTCCTCCTGAC	*Pseudoalteromonas* sp.<br><br>GenBank ID MF061255.1	99 %	0.0
**B3**	GTGGTAACGTCCTCCCGAGGGTTAGACTATCTACTTC<br>TGGAGCAACCCACTCCCATGGTGTGACGGGCGGTGT<br>GTACAAGGCCCGGGAACGTATTCACCGCGTCATTCTG<br>ATACGCGATTACTAGCGATTCCGACTTCATGGAGTCG	*Pseudoalteromonas* sp.<br><br>GenBank ID MF061255.1	99 %	0.0

	AGTTGCAGACTCCAATCCGGACTACGACGCACTTTAA<br>GTGATTCGCTTACCTTCGCAGGTTCGCAGCACTCTGTA<br>TGCGCCATTGTAGCACGTGTGTAGCCCTACACGTAAG<br>GGCCATGATGACTTGACGTCGTCCCCACCTTCCTCCG<br>GTTTATCACCGGCAGTCTCCTTAGAGTTCTCAGCATTA<br>CCTGCTAGCAACTAAGGATAGGGGTTGCGCTCGTTGC<br>GGGACTTAACCCAACATCTCACAACACGAGCTGACGA<br>CAGCCATGCAGCACCTGTATCAGAGTTCCCGAAGGCA<br>CCAAACCATCTCTGGTAAGTTCTCTGTATGTCAAGTGT<br>AGGTAAGGTTCTTCGCGTTGCATCGAATTAAACCACA<br>TGCTCCACCGCTTGTGCGGGCCCCCGTCAATTCATTTG<br>AGTTTTAACCTTGCGGCCGTACTCCCCAGGCGGTCTA<br>CTTAATGCGTTAGCTTTGAAAAACAGAACCGAGGCTC<br>CGAGCTTCTAGTAGACATCGTTTACGGCGTGGACTAC<br>CGGGGTATCTAATCCCGTTTGCTCCCCACGCTTTCGTA<br>CATGAGCGTCAGTGTTGACCCAGGTGGCTGCCTTCGC<br>CATCGGTATTCCTTCAGATCTCTACGCATTTCACCGCT<br>ACACCTGAAATTCTACCACCCTCTATCACACTCTAGTT<br>TGCCAGTTCGAAATGCAGTTCCCAGGTTGAGCCCGGG<br>GCTTTCACATCTCGCTTAACAAACCGCCTGCGTACGCT<br>TTACGCCCAGTAATTCCGATTAACGCTCGCACCCTCCG<br>TATTACCGCGGCTGCTGGCACGGAGTTAGCCGGTGCT<br>TCTTCTGTC			
**B4**	GTAACGTCCTCCCGAGGGTTAGACTATCTACTTCTGG<br>AGCAACCCACTCCCATGGTGTGACGGGCGGTGTGTA<br>CAAGGCCCGGGAACGTATTCACCGCGTCATTCTGATA<br>CGCGATTACTAGCGATTCCGACTTCATGGAGTCGAGT<br>TGCAGACTCCAATCCGGACTACGACGCACTTTAAGTG<br>ATTCGCTTACCTTCGCAGGTTCGCAGCACTCTGTATGC<br>GCCATTGTAGCACGTGTGTAGCCCTACACGTAAGGGC<br>CATGATGACTTGACGTCGTCCCCACCTTCCTCCGGTTT<br>ATCACCGGCAGTCTCCTTAGAGTTCTCAGCATTACCTG<br>CTAGCAACTAAGGATAGGGGTTGCGCTCGTTGCGGG<br>ACTTAACCCAACATCTCACAACACGAGCTGACGACAG<br>CCATGCAGCACCTGTATCAGAGTTCCCGAAGGCACCA<br>AACCATCTCTGGTAAGTTCTCTGTATGTCAAGTGTAG<br>GTAAGGTTCTTCGCGTTGCATCGAATTAAACCACATG<br>CTCCACCGCTTGTGCGGGCCCCCGTCAATTCATTTGA<br>GTTTTAACCTTGCGGCCGTACTCCCCAGGCGGTCTACT<br>TAATGCGTTAGCTTTGAAAAACAGAACCGAGGCTCCG<br>AGCTTCTAGTAGACATCGTTTACGGCGTGGACTACCG<br>GGGTATCTAATCCCGTTTGCTCCCCACGCTTTCGTACA<br>TGAGCGTCAGTGTTGACCCAGGTGGCTGCCTTCGCCA<br>TCGGTATTCCTTCAGATCTCTACGCATTTCACCGCTAC<br>ACCTGAAATTCTACCACCCTCTATCACACTCTAGTTTG<br>CCAGTTCGAAATGCAGTTCCCAGGTTGAGCCCGGGG<br>CTTTCACATCTCGCTTAACAAACCGCCTGCGTACGCTT<br>TACGCCCAGTAATTCCGATTAACGCTCGCACCCTCCGT<br>ATTACCGCGGCTGCTGGCACGGAGTTAGCCGGTGCTT<br>CTT	*Pseudoalteromonas* sp.<br><br>GenBank ID MF061255.1	99 %	0.0

References

1. Boenigk J, Arndt H. Comparative studies on the feeding behavior of two heterotrophic nanoflagellates: the filterfeeding choanoflagellate *Monosiga ovata* and the raptorial-feeding kinetoplastid *Rhynchomonas nasuta*. Mar Ecol Prog Ser. 2000; 22: 243-249.
2. Sherr BF, Sherr EB, Berman T. Grazing, growth, and ammonium excretion rates of a heterotrophic microflagellate fed with four species of bacteria. Appl Environ Microbiol. 1983; 45: 1196-1201.
3. Andersen P. Functional biology of the choanoflagellate *Diaphanoeca grandis* Ellis. Mar Microb Food Webs. 1988/1989; 3: 35-50.
4. Fenchel T. Ecology of heterotrophic microflagellates. II. Bioenergetics and growth. Mar Ecol Prog Ser. 1982; 8: 225-231.
5. Geider RJ, Leadbeater BSC. Kinetics and energetics of growth of the marine choanoflagellate *Stephanoeca diplocostata*. Mar Ecol Prog Ser. 1988; 47: 169-177.
6. Eccleston-Parry JD, Leadbeater BS. A comparison of the growth kinetics of six marine heterotrophic nanoflagellates fed with one bacterial species. Mar Ecol Prog Ser. 1994; 105: 167-177.
7. Hadziavdic K, Lekang K, Lanzen A, Jonassen I, Thompson EM, Troedsson C. Characterization of the 18s rRNA gene for designing universal eukaryote specific primers. PLoS ONE. 2014; 9: e87624.
8. Frank JA, Reich CI, Sharma S, Weisbaum JS, Wilson BA, Olsen GJ. Critical evaluation of two primers commonly used for amplification of bacterial 16S rRNA genes. Appl Environ Microbiol. 2008; 74: 2461–2470.
9. Bratbak G, Dundas I. Bacterial dry matter content and biomass estimations. Appl Environ Microbiol. 1984; 48: 755-757
